# Fibroblast Growth Factor Receptor 4 Deficiency Mediates Airway Inflammation in the Adult Healthy Lung?

**DOI:** 10.3389/fmed.2020.00317

**Published:** 2020-07-24

**Authors:** Molly Easter, Jaleesa Garth, Elex S. Harris, Ren-Jay Shei, Eric S. Helton, Yuhua Wei, Rebecca Denson, Rennan Zaharias, Steven M. Rowe, Patrick Geraghty, Christian Faul, Jarrod W. Barnes, Stefanie Krick

**Affiliations:** ^1^Division of Pulmonary, Allergy and Critical Care Medicine, Department of Medicine, The University of Alabama at Birmingham, Birmingham, AL, United States; ^2^Gregory Fleming James Cystic Fibrosis Research Center, The University of Alabama at Birmingham, Birmingham, AL, United States; ^3^Division of Pulmonary & Critical Care Medicine, Department of Medicine, State University of New York Downstate Medical Center, Brooklyn, NY, United States; ^4^Division of Nephrology and Hypertension, Department of Medicine, The University of Alabama at Birmingham, Birmingham, AL, United States

**Keywords:** fibroblast growth factor receptor 4, lung, inflammation, bronchial epithelium, interleukin 6, airway surface liquid volume

## Abstract

Fibroblast growth factor receptor (FGFR) 4 has been shown to mediate pro-inflammatory signaling in the liver and airway epithelium in chronic obstructive pulmonary disease. In past reports, FGFR4 knockout (*Fgfr4*^−/−^*)* mice did not show any lung phenotype developmentally or at birth, unless FGFR3 deficiency was present simultaneously. Therefore, we wanted to know whether the loss of FGFR4 had any effect on the adult murine lung. Our results indicate that adult *Fgfr4*^−/−^ mice demonstrate a lung phenotype consisting of widened airway spaces, increased airway inflammation, bronchial obstruction, and right ventricular hypertrophy consistent with emphysema. Despite downregulation of FGF23 serum levels, interleukin (IL) 1β and IL-6 in the *Fgfr4*^−/−^ lung, and abrogation of p38 signaling, primary murine *Fgfr4*^−/−^ airway cells showed increased expression of IL-1β and augmented secretion of IL-6, which correlated with decreased airway surface liquid depth as assessed by micro-optical coherence tomography. These findings were paralleled by increased ERK phosphorylation in *Fgfr4*^−/−^ airway cells when compared with their control wild-type cells. Analysis of a murine model with constitutive activation of FGFR4 showed attenuation of pro-inflammatory mediators in the lung and airway epithelium. In conclusion, we are the first to show an inflammatory and obstructive airway phenotype in the adult healthy murine *Fgfr4*^−/−^ lung, which might be due to the upregulation of ERK phosphorylation in the *Fgfr4*^−/−^ airway epithelium.

## Introduction

Fibroblast growth factor receptors (FGFRs), a subfamily of receptor tyrosine kinases, consist of four family members, including FGFR1, 2, 3, and 4 (1). Dependent on different FGF ligands, FGFRs exert diverse functions through the activation of downstream signaling pathways. In organs that are contributing to phosphate homeostasis such as the kidney and parathyroid gland, signaling occurs through FGFR1 by binding FGF23 and its co-receptor klotho (2, 3). In organs that do not express klotho, or in a state of klotho deficiency, FGF23 can activate FGFR4 and induce the phosphorylation of phospholipase γ (PLCγ), leading to hypertrophic growth in cardiac myocytes and the production of inflammatory cytokines in the liver (4, 5). FGFR3 and FGFR4 are abundantly expressed in both the epithelium and mesenchyme in the developing mammalian lung (6–8). Weinstein et al. generated mice homozygous for a deletion of *Fgfr4*; however, these mice did not exhibit any overt abnormalities in the lungs or other organs after birth. Mice homozygous for targeted disruption of both *Fgfr3* and *Fgfr4* had lungs that were completely blocked in alveologenesis without formation of secondary septae (9).

Bronchopulmonary dysplasia, a pulmonary disorder of the newborn and associated with hyperoxia-induced lung injury, is also significantly linked with a polymorphism in the FGFR4 gene (9, 10). An established murine bronchopulmonary dysplasia model also demonstrated decreased expression of *Fgfr3* and *Fgfr4* (11). In addition, inactivation of both *Fgfr3* and *Fgfr4* in the embryonic mouse lung mesenchyme, but not the epithelium, led to a defect in elastogenesis with upregulation of Mfap5, the gene encoding the extracellular matrix component MAGP-2, a critical elastic component of extracellular matrix microfibrils (12). In the adult lung, FGFR4 is overexpressed in non-small cell lung cancer and can induce proliferation (13).

We have recently shown that FGFR4 signaling is also activated in chronic obstructive pulmonary disease (COPD) (14). Human bronchial epithelial cells from COPD patients, which were differentiated at the air–liquid interface, showed an increased expression of FGFR4, which seemed to mediate secretion of interleukin (IL) 1β via activation of PLCγ/nuclear factor of activated T-cells. Inhibition of FGFR4 attenuated this effect, implying a pathological role for FGFR4 in the COPD lung. However, there is not much known about the effect of FGFR4 deficiency in the healthy adult lung. The loss of FGFR4 has been shown to affect metabolism; Huang et al. showed that *Fgfr4*^−/−^ mice developed features of the metabolic syndrome, including an increase in white adipose tissue mass, insulin resistance, glucose intolerance, and hyperlipidemia, when challenged with a high-fat diet (15). This report aims to analyze the physiological role of FGFR4 in the adult lung. Based on our recent findings, we initially hypothesized that FGFR4 deficiency is protective against airway inflammation.

## Materials and Methods

### Study Approval

All animal protocols were approved by the Institutional Animal Care and Use Committees at the University of Alabama at Birmingham (UAB). Both *Fgfr4*^−/−^ and *Fgfr4-Arg/Arg385* mouse models were provided by Dr. Christian Faul. Both mouse models were generated on the C57BL/6 background, as previously described (4). All animals were housed and bred in UAB facilities that were accredited by the Association for Assessment and Accreditation of Laboratory Animal Care International (AAALAC), where they were under the supervision of a team of veterinarians and staff. They were monitored daily by the investigators. UAB complies with the National Institutes of Health policies on animal welfare, the Animal Welfare Act, and all other applicable federal, state, and local laws.

### Primary Murine Tracheal Epithelial Cell Cultures

Primary murine tracheal epithelial cells (MTECs) were isolated from *Fgfr4*^+/+^, *Fgfr4*^−/−^, and *Fgfr4-Gly/Gly385 (control) Fgfr4-Arg/Arg385 mouse tracheas* and plated on collagen IV-coated clear 12-mm-transwell filters (Corning, Corning, NY). MTECs were cultured and some of them differentiated for 2–4 weeks at the air–liquid interface (ALI) as described previously (14).

### Western Blot

All protein lysates were obtained from MTECs and murine lungs using radioimmunoprecipitation assay buffer with phosphatase and protease inhibitors. Proteins were separated on 4–20% precast Ready Gels (Bio-Rad, Hercules, CA, USA) and transferred onto polyvinylidene difluoride (PVDF) membranes (Pierce, Thermo Fisher Scientific, Waltham, MA, USA). Membranes were blocked with 5% low-fat milk in Tris-buffered saline (pH 7.4) with 0.05% Tween 20 (TBST) for 30 min and incubated overnight with the following primary antibodies: rabbit anti-FGFR4, rabbit total and phospho-anti-ERK1/2, rabbit total and phospho-anti-p38 mitogen-activated protein kinase (MAPK), rabbit total and phospho-anti-PLCγ1 (Cell Signaling Technologies, Danvers, MA, USA), and mouse anti-β-actin-peroxidase (Sigma, St. Louis, MO, USA). After five washes with TBST, membranes were incubated with a goat anti-rabbit peroxidase conjugated (Invitrogen) at 1:5,000 in TBST for 45 min. Positive signals were visualized by chemiluminescence on a ChemiDoc XRS system (Bio-Rad). Images were acquired using Image Lab software (Bio-Rad). Densitometry was measured using ImageJ software (National Institutes of Health).

### Enzyme-Linked Immunosorbent Assay

An enzyme-linked immunosorbent assay (ELISA) for the quantitative detection of mouse interleukin (IL) 6 (Invitrogen, Vienna, Austria) was used on the basolateral medium from MTECs. IL-6 protein levels in the basolateral medium from MTECs ranged from 25 to 1,500 pg/ml. We had one outlier ALI culture, which showed IL-6 protein levels of >3,000 pg/ml, which was excluded after statistical outlier analysis.

### RNA Extraction and Quantitative Real-Time PCR

Total RNA was extracted from MTECs and murine lungs, as previously described (16). Real-time quantitative PCRs were performed using the following TaqMan probes: Fgfr4 Mm00433314, Fgf23 Mm00445621, Il-6 Mm00446190, Il-1beta Mm00434228, Tgf-beta1 Mm01178820, and Gapdh Mm99999915 (Invitrogen, Carlsbad, CA, USA).

### Micro-Optical Coherence Tomography

Micro-optical coherence tomography imaging was performed on differentiated MTECs using the same technique as previously described (17, 18).

### Analysis of Lung Function in Mice

Six-month-old mice were anesthetized, and pulmonary function was evaluated using the ratio of forced expiratory volume/forced vital capacity (FEV0.5/FVC) as a measure for airway obstruction and quasi-static compliance (Cst) with a flexiVent (SCIREQ; Montreal, Canada) as described previously (19).

### Bronchoalveolar Lavage

Bronchoalveolar lavage (BAL) fluid was collected as previously described (20). Briefly, 1 ml of phosphate-buffered saline was instilled via a tracheal cannula into the lungs and aspirated. The collected BAL fluid was then centrifuged at 500 g for 5 min at 4°C to pellet the cells. The cell pellet was then resuspended in 100–150 μl of phosphate-buffered saline to determine the total cell count. Cytospins were prepared as previously described, and Wright–Giemsa staining for differential cell counts was performed using the HEMA 3 Stain Set (Fisher Scientific, Hampton, NH, USA) as directed by the manufacturer (14).

### Lung Histology and Morphometric Analysis

Mice were euthanized and perfused via the right ventricle with 3 ml of phosphate-buffered saline. The lungs were inflated using 1 ml of 10% neutral buffered formalin and fixed for 24 h. After the lungs were dehydrated in 70% ethanol, they were processed and embedded in paraffin. Sections were cut 3–5 mm, mounted on slides, and stained with hematoxylin. Anti-rabbit FGFR4 antibody (sc-124; Santa Cruz Biotechnology, Dallas, TX, USA) was used for staining total lung tissue embedded in paraffin. Morphometric analysis was performed assessing mean linear intercepts after fixation of the murine lung tissue, as previously described in detail (19, 21).

### Statistics

Data were analyzed with Prism5 (GraphPad Software, Inc., La Jolla, CA) and shown as mean ± SEM using Student's *t*-test and analysis of variance or the Kruskal–Wallis test with appropriate posttests for at least three independent experiments. Significance was accepted at *p* < 0.05.

## Results

### Fgfr4 Deficiency in Mice Leads to an Airway Phenotype Resembling Chronic Obstructive Pulmonary Disease

**Figure 1:**
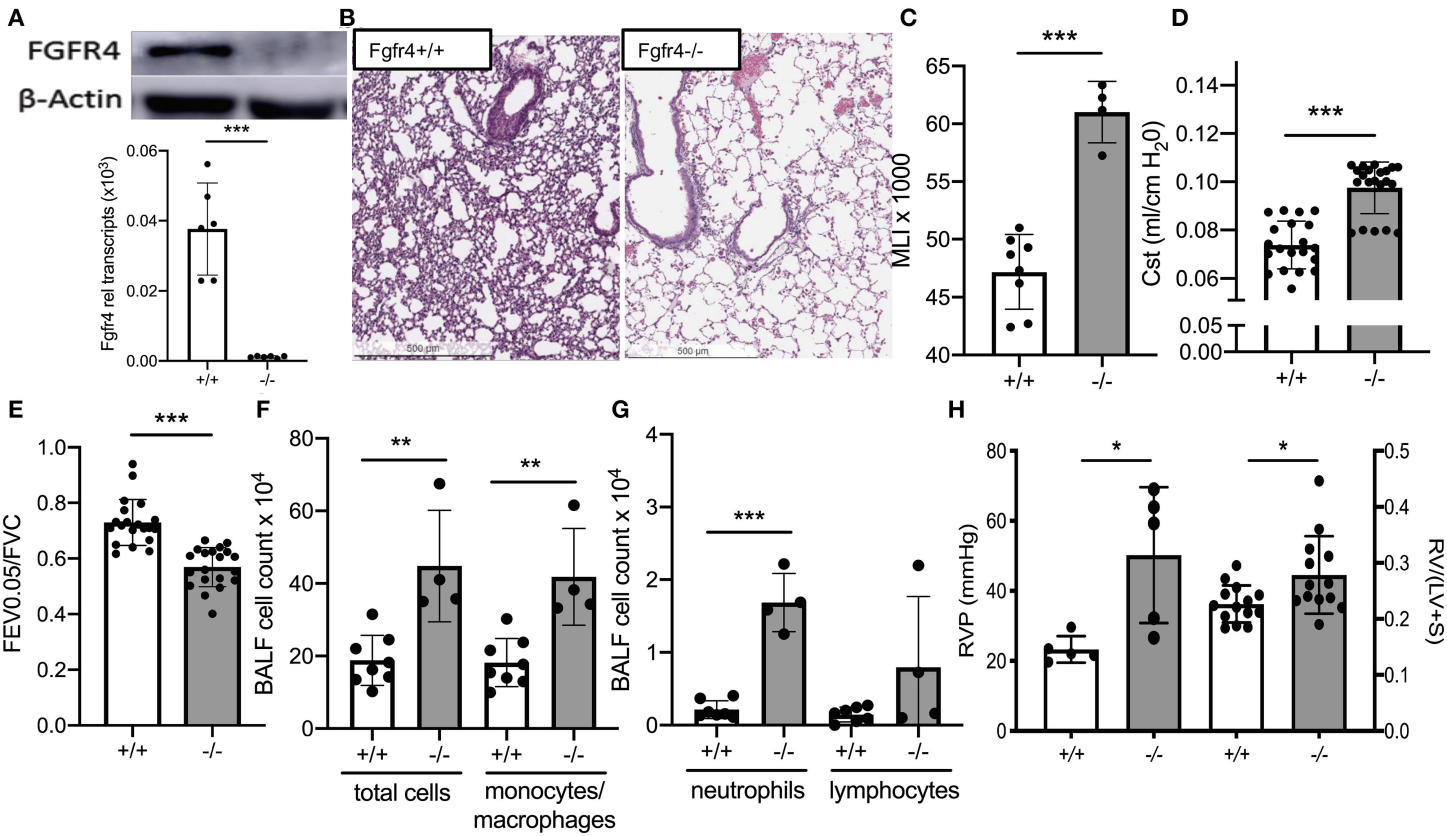
*In vivo effect of FGFR4 deficiency*. **(A)** Immunoblot using an anti-FGFR4 antibody (upper image) and bar graphs showing Fgfr4 mRNA levels (lower diagram) in whole lung tissue from *Fgfr4*^−/−^ and *Fgfr4*^+/+^ mice. **(B)** Immunohistochemistry using hematoxylin staining of paraffin-embedded lung tissue (20 ×) demonstrating widened airway spaces in *Fgfr4*^−/−^ mice. **(C)** Bar graphs showing morphometric analysis (mean linear intercepts) of lung tissue from *Fgfr4*^−/−^ and *Fgfr4*^+/+^ mice. **(D)** Quasi-static compliance (Cst) and **(E)** ratio of forced expiratory volume (FEV) and forced ventilatory capacity (FVC), assessed by flexiVent. **(F)** Bar graphs showing total cell count, total macrophage, monocyte count, and **(G)** total neutrophil and lymphocyte count from bronchoalveolar lavage fluid. **(H)** Bar graphs indicating hemodynamic analysis of right ventricular pressure and Fulton index (RV/LV+S) of *Fgfr4*^−/−^ and *Fgfr4*^+/+^ hearts. Statistical analysis was done using ANOVA or Student's *t*-test showing means ± SEM with *p < 0.05, **p < 0.01, and ***p < 0.001 with *n* = 4–15 mice per group.

We analyzed the lungs of 6-month-old *Fgfr4*^−/−^ mice and compared them to their age-matched wild-type littermates. We did not detect any FGFR4 protein or messenger RNA (mRNA) expression in *Fgfr4*^−/−^ lungs (Figure 1A). Interestingly, paraffin-embedded slides from murine *Fgfr4*^−/−^ lungs revealed airway enlargement (Figure 1B), which was reflected in a significant increase in mean linear intercepts (Figure 1C). Using flexiVent to analyze lung function, *Fgfr4*^−/−^ lungs showed a significant increase in static compliance (Figure 1D) and a marked decrease in the FEV0.05/FVC ratio (Figure 1E), indicating airway obstruction. In addition, there was a significant increase in the total cell count, monocytes/macrophages, and neutrophils in the BAL fluid without any difference in the lymphocyte count (Figures 1F,G). Collectively, full *Fgfr4*^−/−^ mice at 6 months of age showed evidence of airway inflammation and airway enlargement consistent with emphysema.

### Fgfr4 Deficiency in Mice Affects the Right Heart

In addition to the inflammatory lung phenotype, 6-month-old *Fgfr4*^−/−^ mice demonstrated an increase in right ventricular pressure as assessed by right heart catheterization and evidence of right ventricular hypertrophy as assessed by the Fulton index, when compared with their age-matched littermates (Figure 1H).

### Fgfr4 Deficiency Affects Fibroblast Growth Factor 23 Levels and Inflammation and Abrogates Phosphorylation of p38 in Murine Lungs

**Figure 2:**
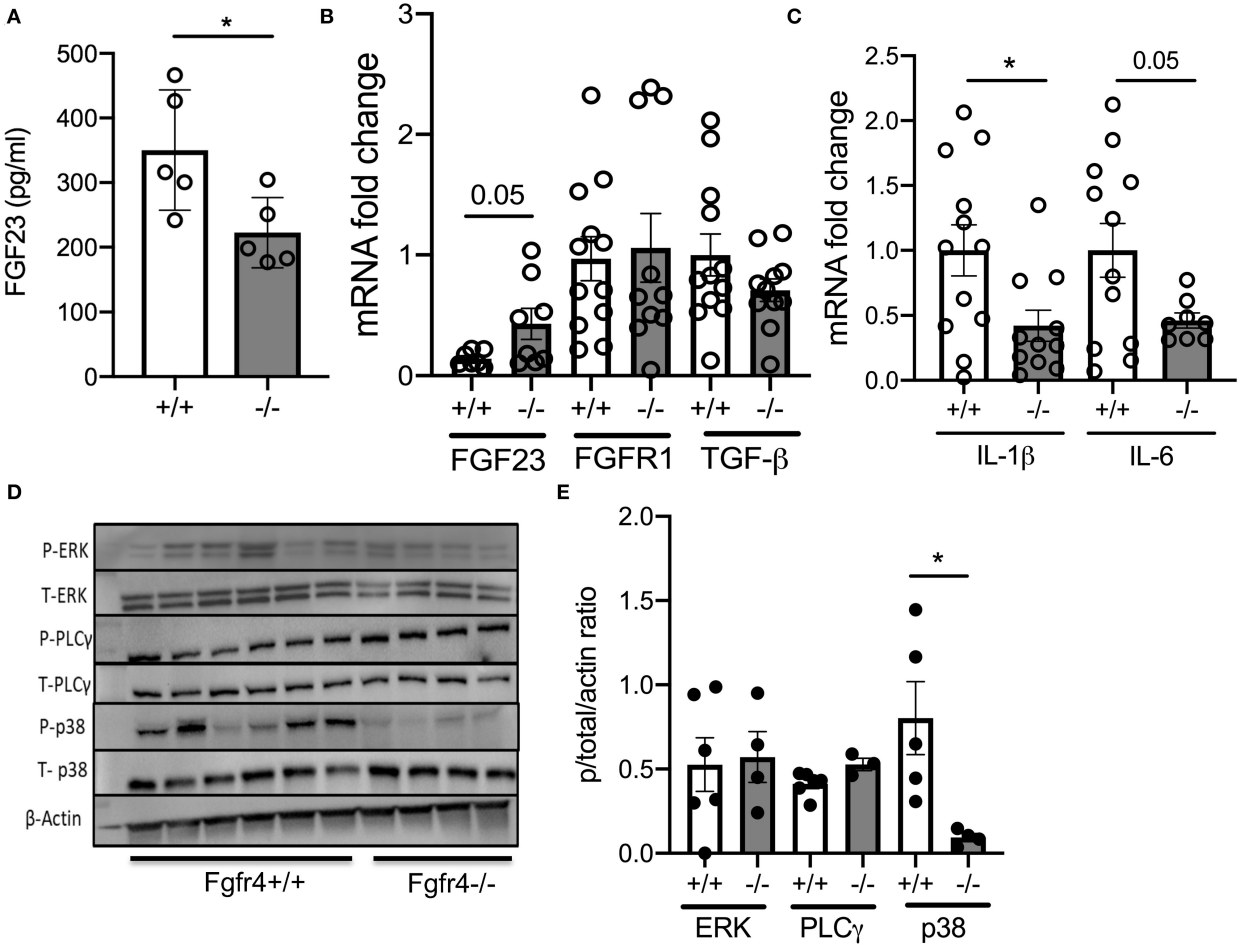
*Effect of FGFR4 deficiency in the murine lung*. **(A)** Bar graphs indicating FGF23 serum levels from *Fgfr4*^−/−^ and *Fgfr4*^+/+^ mice. **(B)** mRNA fold change in FGF23, FGFR1, and TGF-β levels and **(C)** pro-inflammatory mediators such as IL-1β and IL-6 in total lung tissue from *Fgfr4*^−/−^ and *Fgfr4*^+/+^ mice. **(D)** Representative immunoblot analyses and **(E)** quantification by densitometry of phospho-ERK, total ERK, phospho-PLCγ, total PLCγ, phospho-p38, and total p38 from total lung tissue of *Fgfr4*^−/−^ and *Fgfr4*^+/+^ mice. (All *n* = 3–6 mice per group showing mean ± SEM with *P < 0.05).

Interestingly, *Fgfr4*^−/−^ mice had significantly decreased FGF23 serum levels (Figure 2A) but increased expression of FGF23 mRNA in *Fgfr4*^−/−^ lungs without any changes in FGFR1 or transforming growth factor (TGF) β mRNA levels (Figure 2B). Furthermore, IL-1β and IL-6 mRNA levels were downregulated by 2-fold in total lung lysates from *Fgfr4*^−/−^ mice (Figure 2C). Analysis of potentially affected signaling pathways revealed that *Fgfr4*^−/−^ lungs showed a negligible change in phosphorylation of PLCγ but an almost complete abrogation of p38 phosphorylation and downregulation of both phospho-ERK and total ERK expression (Figures 2D,E).

### Fibroblast Growth Factor Receptor 4 Deficiency Affects Mucociliary Clearance and Inflammation in Murine Tracheal Epithelial Cells

**Figure 3:**
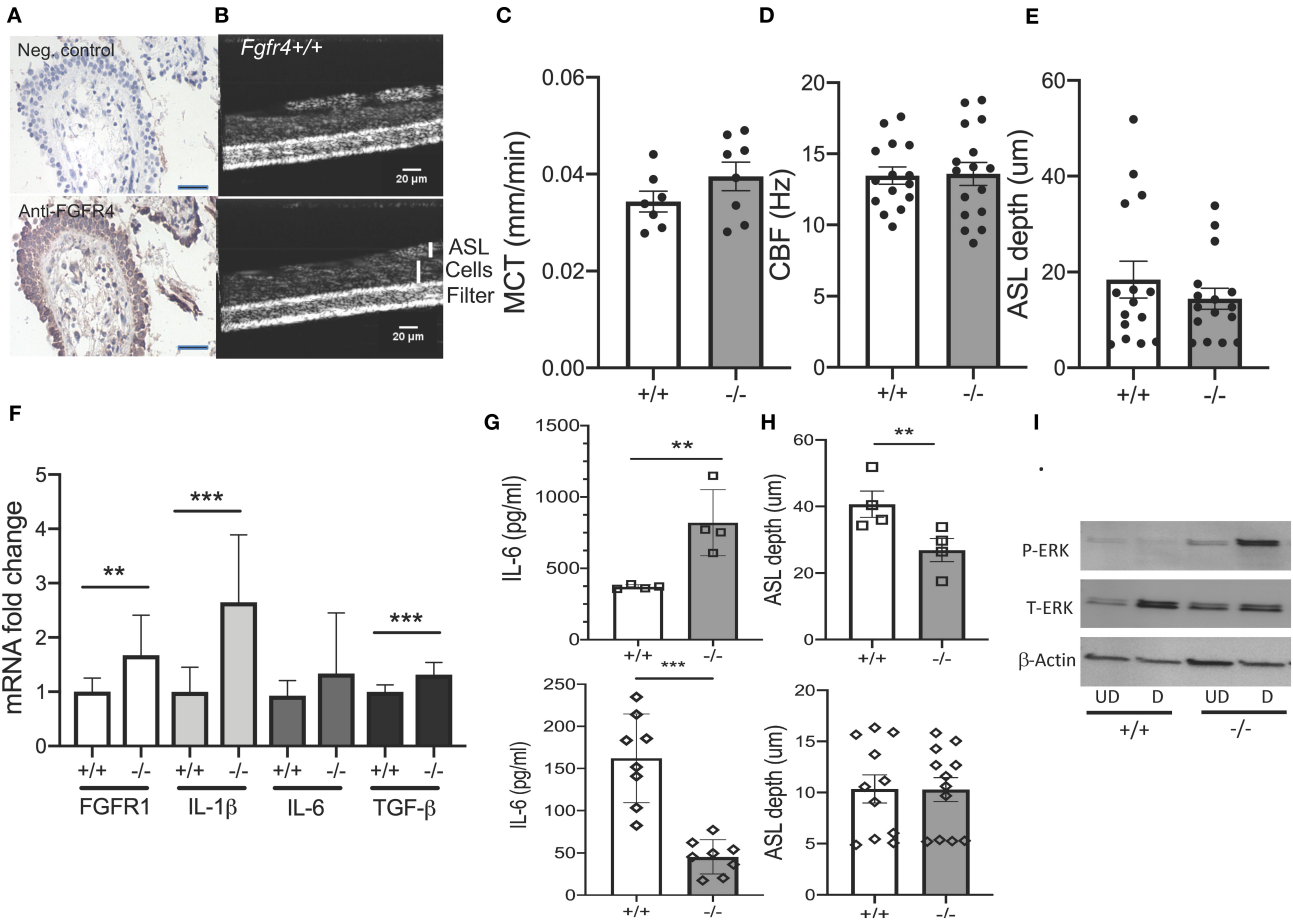
*Effect of FGFR4 deficiency in the murine airway epithelium*. **(A)** Immunohistochemistry using anti-IgG (negative control) (upper image) and anti-FGFR4 antibody (lower image) and counter hematoxylin staining in a representative human lung section showing staining of the airway epithelium (arrows). **(B)** Representative images of *Fgfr4*^−/−^ and *Fgfr4*^+/+^ MTECs, differentiated on filters using micro-optical coherence tomography. **(C)** Quantification of mucociliary transport (MCT), **(D)** ciliary beat frequency (CBF), and **(E)** airway surface liquid (ASL) depth from differentiated *Fgfr4*^−/−^ and *Fgfr4*^+/+^ MTECs. **(F)** mRNA fold changes of FGFR1, IL-1β, IL-6, and TGF- β levels in *Fgfr4*^−/−^ and *Fgfr4*^+/+^ MTECs. **(G)** IL-6-protein levels, assessed in supernatant from *Fgfr4*^−/−^ and *Fgfr4*^+/+^ MTECs, when divided into different isolation groups and **(H)** their respective ASL depth analysis. **(I)** Representative immunoblot analyses of undifferentiated (UD) and differentiated (D) *Fgfr4*^−/−^ and *Fgfr4*^+/+^ MTECs assessing expression of phospho-ERK, total ERK, and β-actin. (Three separate experiments from three to six mice per group. All bar graphs are mean ± SEM with **P < 0.01, and ***P < 0.005).

To further elucidate the role of FGFR4 in the airway epithelium (Figure 3A) (14), mucociliary clearance was assessed in primary MTECs from *Fgfr4*^−/−^ mice and their wild-type littermates. MTECs were differentiated at the ALI and mucociliary clearance parameters were analyzed by micro-optical coherence tomography (Figure 3B). There was no difference in mucociliary transport, ciliary beat frequency, and airway surface liquid (ASL) depth between wild-type and *Fgfr4*^−/−^ MTECs (Figures 3C–E). We did note, however, an increase in FGFR1, IL-1β, and TGF-β mRNA levels in *Fgfr4*^−/−^ MTECs when compared with those in MTECSs from wild-type littermates (Figure 3F). MTECs did not show expression of FGF23 (data not shown). Analysis of IL-6 protein levels in cell culture media did not show any significant difference between *Fgfr4*^−/−^ and *Fgfr4*^+/+^ MTECs when data were pooled (not shown) but showed significant differences when substratified according to the different isolations. MTEC isolations with higher IL-6 levels showed a further increase in the *Fgfr4*^−/−^ cultures (Figure 3G, upper graph), which was reversed in the MTEC isolations with lower IL-6 protein levels (Figure 3G, lower graph). MTECs were isolated from three to six *Fgfr4*^+/+^ and *Fgfr4*^−/−^ mice and pooled, and half of them were plated submerged (undifferentiated); the other half were differentiated at the ALI for 3–4 weeks—these were used for ASL measurements. We compared the differentiated MTECs of the same isolation with their respective *Fgfr4*^+/+^ control MTECs. Post-stratification analysis of ASL depth revealed that the “high IL-6 secretor group” showed a significantly decreased ASL depth (Figure 3H, upper graph), whereas there was no difference in ASL depth in the “low IL-6 secretor group” (Figure 3H, lower graph). Analysis of potentially involved signaling pathways in both undifferentiated (UD) and differentiated (D) MTECs revealed an upregulation of phospho-ERK (Figure 3I).

In summary, MTECs isolated from *Fgfr4*^−/−^ mice demonstrated heterogeneity in IL-6 protein levels but did not exhibit any significant differences in mucociliary clearance. Interestingly, *Fgfr4*^−/−^ MTEC cultures with high IL-6 protein secretion showed a decrease in ASL depth and increased phosphorylation of ERK.

### Constitutive Fibroblast Growth Factor Receptor 4 Activation Inhibits Expression of Inflammatory Mediators in Murine Lungs and Tracheal Epithelial Cells

**Figure 4:**
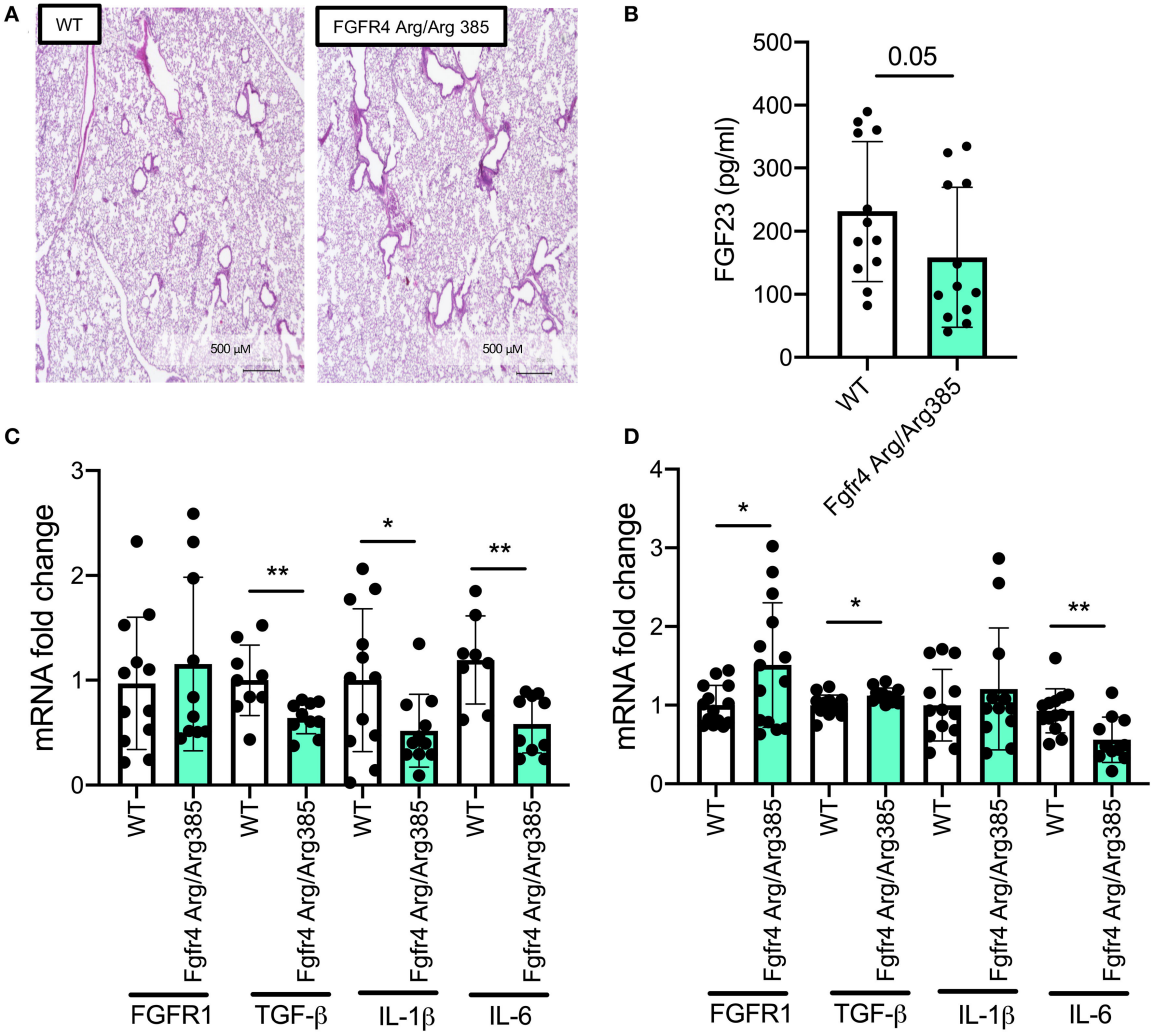
*Effect of constitutive FGFR4 activation in the murine lung and airway epithelium*. **(A)** Hematoxylin staining in representative murine lung sections of control and *Fgfr4-Arg/Arg385* mice. **(B)** Bar graphs indicating serum Fgf23 levels and **(C)** fold changes of FGFR1, TGF-β, IL-1β, and IL-6 mRNA levels in total lung tissue and **(D)** MTECs, isolated from *Fgfr4-Arg/Arg385* mice and their wild-type littermates. (Three separate experiments from three to six mice per group. All bar graphs are mean ± SEM with *P < 0.05 and **P < 0.01).

As *Fgfr4*^−/−^ lungs show airway inflammation and airway enlargement consistent with emphysema, we wanted to assess the effect of constitutive FGFR4 activation. Therefore, we analyzed lung tissue and MTEC cultures from *Fgfr4-Arg/Arg385* mice. This knockin mouse model exhibits a gain-of-function mutation in *Fgfr4*, replacing a glycine with an arginine residue in the transmembrane domain, leading to increased FGFR4 stability and prolonged phosphorylation (4, 22). Microscopic assessment of the pulmonary tissue of *Fgfr4-Arg/Arg385* mice did not show any differences compared with that of wild-type littermates (Figure 4A). Circulating FGF23 plasma levels were slightly decreased in *Fgfr4-Arg/Arg385* mice (Figure 4B). mRNA analysis of lung tissue demonstrated significant decreases in inflammatory mediators such as TGF-β, IL-1β, and IL-6 (Figure 4C), whereas *Fgfr4-Arg/Arg385* MTECs showed significant increases in FGFR1 and a significant decrease in IL-6 mRNA expression (Figure 4D). In summary, *Fgfr4-Arg/Arg385* lungs did not show any airway or parenchymal changes, and inflammatory mediator expression was downregulated in MTECs and total lungs when compared with age-matched MTECs/lungs from wild-type littermates.

## Discussion

This report is the first to characterize and demonstrate a cardiopulmonary phenotype, including emphysema, airway inflammation, and right ventricular hypertrophy, in the adult *Fgfr4*^−/−^ mouse. *Fgfr4*^−/−^ lungs showed airway inflammation with upregulation of neutrophils and monocytes/macrophages in the BAL fluid and upregulation of inflammatory mediators in airway cells showing an association of high IL-6 and decreased ASL depth. When trying to decipher a potential pathway, interestingly, ERK phosphorylation did not seem to be affected in whole lungs, whereas there was a complete abrogation of p38 phosphorylation in the *Fgfr4*^−/−^ lung.

PLCγ-1 is expressed abundantly in macrophages and can be activated by FGFs and also other growth factors, including platelet-derived growth factor (PDGF), vascular endothelial growth factor (VEGF), and epidermal growth factor (23). Furthermore, TNF-α and IL-1β were also identified as upstream stimulators for PLCγ-1 (24, 25). The influenza virus H1N1 infection can activate PLCγ-1 via the epidermal growth factor receptor in alveolar epithelial cells (25). Therefore, an increase in airway inflammation might be partially due to FGFR4-independent activation of PLCγ, as our data showed persistent phosphorylation of PLCγ, even in the *Fgfr4*^−/−^ lungs. Interestingly, there was a complete abrogation of p38 phosphorylation in the FGFR4 deficient lungs. It has been shown that p38 MAPK pathways play a key role in the inflammatory response in sepsis-induced acute lung injury via lipopolysaccharide-induced TLR-4 signaling (26, 27). This activation leads to IL-8 production in human mononuclear cells (28). A similar pathway has been shown in the bronchial epithelium, where lipopolysaccharide inhalation preceded the increased expression of p38 MAPK and IL-8 (29). p38 MAPK activation has also been shown to induce histone modifications leading to increased accessibility for transcription factors to promotor regions and enhanced inflammatory gene expression (30). In COPD lungs, there is an increase in p38 phosphorylation (31), and p38 MAPK inhibitors might be useful in targeting airway inflammation by reducing cytokine production of alveolar macrophages (20, 32, 33).

These findings contradict our results that FGFR4 deficiency and an abrogated p38 response seemed to be involved in triggering airway inflammation. One explanation might be that the downregulation of p38 could paradoxically suppress an anti-inflammatory effect, as shown in rheumatoid arthritis (34). Another potential mechanism could involve ERK–p38 crosstalk, which may lead to prolonged activation of FGFR1 (35); this could lead to inflammation. It was also very surprising that the analysis of pro-inflammatory cytokines revealed downregulation in the lung tissue but upregulation in MTECs, which could be due to the activation of compensatory anti-inflammatory pathways in pulmonary cells other than the bronchial epithelium. Finally, ALI cultures are often used to model regeneration and repair (36); hence, it may be linked to benefits or pathologies when *Fgfr4*^−/−^ mice repair their airways and lungs from stressors. Future experiments exposing *Fgfr4*^−/−^ mice to cigarette smoke short and long term or expose them to respiratory viruses such as influenza or rhinovirus, two known COPD exacerbating viruses, would help to explain further the role and potential mechanism of FGFR4 in airway inflammation.

When specifically assessing the airway epithelium with our *in vitro* studies, there was no significant difference in mucociliary clearance parameters, which could be explained by the lack of interaction with inflammatory cells in our ALI cultures, which differs from our *in vivo* results. In addition, MTEC cultures were quite heterogeneous in regard to their baseline ASL volumes, and relatively “dry” MTECs overall did not show significant differences. Interestingly, *Fgfr4*^−/−^ MTECs, which had high secretion of IL-6 protein, also had significantly decreased ASL depth. Immunoblot also revealed increased ERK phosphorylation in these cultures as a potential underlying pro-inflammatory mechanism supporting this hypothesis of ERK–p38 crosstalk. To associate our outcomes with FGFR4 deficiency, we phenotypically analyzed the lungs of FGFR4 “knockin” mice. Interestingly, both lung tissue and MTEC cultures showed a decrease in inflammatory mediators.

Overall, we show for the first time that not only FGFR4 overexpression has pathological effects in diseases such as cancer and COPD, but also FGFR4 deficiency in the “healthy” adult lung can lead to airway inflammation. This was not observed in previous reports, which may be because murine lungs were characterized in detail during the embryonic phase and the first weeks in life. Weinstein et al. reported that *Fgfr4*^−/−^ mice do not have a decreased life expectancy and exhibited apparently normal growth, but they weighed 10% less than their wild-type littermates at weaning. *Fgfr4*^−/−^ mice were able to reproduce and did not display any developmental irregularities until 1 year of age, although murine lungs were not characterized in further detail except that they lacked expression of FGFR4 (7). Therefore, FGFR4 deficiency could have been functionally compensated, e.g., downregulation of p38 as an attempt to attenuate inflammation until a second stressor hit, which may be related to aging with a general increase inflammation, hereby exaggerated by FGFR4 deficiency.

To further decipher the role of FGFR4 in the bronchial epithelium, we also analyzed the *Fgfr4-Arg/Arg385* mouse model, which *per se* did not have an airway phenotype. The lung demonstrated decreased inflammatory mediators in the airway. Therefore, it will be very interesting to investigate the role of pathological airway challenges such as cigarette smoke or other air pollutants in the airways in these mice.

This study demonstrates that global FGFR4 deficiency can cause airway pathology and right ventricular hypertrophy in the adult mouse, thereby indicating that FGFR4 signaling in the lung is essential and not only harmful as we have shown in preexisting airway diseases such as COPD (14). Interestingly, the FGF23/FGFR4 axis has been extensively studied in the myocardium, and when challenged by additional chronic kidney disease or high-phosphate diet, *Fgfr4*^−/−^ mice also demonstrate left ventricular hypertrophy. Therefore, further studies are needed to delineate potential crosstalk between heart and lung in diverse pathologies.

In summary, FGFR4 signaling is very complex *in vivo* because in pathological states such as COPD, it can contribute to the pathology, but in the healthy adult lung, FGFR4 seems to exert an anti-inflammatory role in the airway. More studies are needed to decipher the exact signaling pathway(s) as to how FGFR4 contributes to these airway phenotypes.

## Data Availability Statement

The raw data supporting the conclusions of this article will be made available by the authors, without undue reservation.

## Ethics Statement

The animal study was reviewed and approved by The Institutional Animal Care and Use Committee, The University of Alabama at Birmingham.

## Author Contributions

SK, JB, and ME contributed to the concept and/or design of the study. ME, JG, EHa, R-JS, EHe, YW, RD, RZ, SR, PG, CF, JB, and SK contributed to the acquisition of the data. ME, JG, PG, EHa, R-JS, JB, CF, and SK contributed to the analysis and interpretation. ME, JG, JB, and SK drafted the manuscript. All authors critically revised it for intellectual content and approved the final version prior to submission.

## Conflict of Interest

The authors declare that the research was conducted in the absence of any commercial or financial relationships that could be construed as a potential conflict of interest.
